# Supplementary material for: Heparin-based hydrogel scaffolding alters the transcriptomic profile and increases the chemoresistance of MDA-MB-231 triple-negative breast cancer cells
Source: Biomater Sci. 2020 Feb 13;8(10):2786–96. doi: 10.1039/c9bm01481k (PMC7497406; doi:10.1039/c9bm01481k)
Supplement: Supplementary file 2 [file BM-008-C9BM01481K-s002.zip › Supplementary File 4/HP-BhydrogelvControl/Pathways/my_analysis.Gsea.1545201079907/HALLMARK_IL6_JAK_STAT3_SIGNALING.html]

Details for gene set HALLMARK\_IL6\_JAK\_STAT3\_SIGNALING[GSEA]

|  || Dataset | expr.class.cls#HYD\_versus\_CONTROL.class.cls#HYD\_versus\_CONTROL\_repos |
| Phenotype | class.cls#HYD\_versus\_CONTROL\_repos |
| Upregulated in class | CONTROL |
| GeneSet | HALLMARK\_IL6\_JAK\_STAT3\_SIGNALING |
| Enrichment Score (ES) | -0.26377326 |
| Normalized Enrichment Score (NES) | -1.0730883 |
| Nominal p-value | 0.29850745 |
| FDR q-value | 0.32299733 |
| FWER p-Value | 0.965 |
Table: GSEA Results Summary

  

Fig 1: Enrichment plot: HALLMARK\_IL6\_JAK\_STAT3\_SIGNALING      
 Profile of the Running ES Score & Positions of GeneSet Members on the Rank Ordered List

  

| PROBE | DESCRIPTION (from dataset) | GENE SYMBOL | GENE\_TITLE | RANK IN GENE LIST | RANK METRIC SCORE | RUNNING ES | CORE ENRICHMENT || 1 | TYK2 | na |  |  | 940 | 1.916 | 0.0038 | No |
| 2 | MAP3K8 | na |  |  | 1059 | 1.833 | 0.0483 | No |
| 3 | CSF2RA | na |  |  | 1803 | 1.441 | 0.0493 | No |
| 4 | JUN | na |  |  | 1992 | 1.366 | 0.0772 | No |
| 5 | TNFRSF12A | na |  |  | 3645 | 0.883 | 0.0153 | No |
| 6 | STAT2 | na |  |  | 4243 | 0.764 | 0.0052 | No |
| 7 | SOCS3 | na |  |  | 4653 | 0.684 | 0.0027 | No |
| 8 | IRF1 | na |  |  | 5648 | 0.533 | -0.0345 | No |
| 9 | TNFRSF1B | na |  |  | 5672 | 0.529 | -0.0211 | No |
| 10 | IL17RB | na |  |  | 6523 | 0.413 | -0.0541 | No |
| 11 | IL3RA | na |  |  | 6563 | 0.408 | -0.0449 | No |
| 12 | IL17RA | na |  |  | 6787 | 0.385 | -0.0459 | No |
| 13 | CXCL10 | na |  |  | 6890 | 0.372 | -0.0410 | No |
| 14 | CXCL3 | na |  |  | 7031 | 0.355 | -0.0385 | No |
| 15 | PTPN2 | na |  |  | 7106 | 0.347 | -0.0328 | No |
| 16 | IL6 | na |  |  | 7244 | 0.333 | -0.0307 | No |
| 17 | TNF | na |  |  | 7258 | 0.331 | -0.0223 | No |
| 18 | LTB | na |  |  | 7357 | 0.318 | -0.0186 | No |
| 19 | CXCL1 | na |  |  | 7814 | 0.270 | -0.0350 | No |
| 20 | PIM1 | na |  |  | 8219 | 0.229 | -0.0497 | No |
| 21 | IL7 | na |  |  | 8303 | 0.219 | -0.0480 | No |
| 22 | BAK1 | na |  |  | 9101 | 0.141 | -0.0858 | No |
| 23 | IL1B | na |  |  | 9136 | 0.136 | -0.0838 | No |
| 24 | IL4R | na |  |  | 9138 | 0.135 | -0.0801 | No |
| 25 | TNFRSF21 | na |  |  | 9191 | 0.127 | -0.0793 | No |
| 26 | LTBR | na |  |  | 9299 | 0.117 | -0.0817 | No |
| 27 | SOCS1 | na |  |  | 9324 | 0.113 | -0.0798 | No |
| 28 | IL15RA | na |  |  | 9909 | 0.053 | -0.1089 | No |
| 29 | TGFB1 | na |  |  | 9937 | 0.048 | -0.1090 | No |
| 30 | EBI3 | na |  |  | 10141 | 0.023 | -0.1189 | No |
| 31 | CRLF2 | na |  |  | 10407 | 0.006 | -0.1326 | No |
| 32 | PTPN1 | na |  |  | 10546 | -0.009 | -0.1396 | No |
| 33 | CD44 | na |  |  | 11081 | -0.075 | -0.1654 | No |
| 34 | PDGFC | na |  |  | 11304 | -0.090 | -0.1745 | No |
| 35 | TNFRSF1A | na |  |  | 11403 | -0.101 | -0.1768 | No |
| 36 | CD14 | na |  |  | 11546 | -0.118 | -0.1810 | No |
| 37 | MYD88 | na |  |  | 12113 | -0.189 | -0.2054 | No |
| 38 | FAS | na |  |  | 12828 | -0.261 | -0.2355 | No |
| 39 | TLR2 | na |  |  | 12836 | -0.262 | -0.2286 | No |
| 40 | LEPR | na |  |  | 12890 | -0.271 | -0.2239 | No |
| 41 | ACVR1B | na |  |  | 13007 | -0.284 | -0.2221 | No |
| 42 | IL1R1 | na |  |  | 13023 | -0.288 | -0.2149 | No |
| 43 | CSF2 | na |  |  | 13029 | -0.288 | -0.2072 | No |
| 44 | CXCL11 | na |  |  | 13037 | -0.289 | -0.1996 | No |
| 45 | HAX1 | na |  |  | 13045 | -0.290 | -0.1920 | No |
| 46 | PTPN11 | na |  |  | 13303 | -0.326 | -0.1964 | No |
| 47 | IRF9 | na |  |  | 13588 | -0.367 | -0.2011 | No |
| 48 | OSMR | na |  |  | 14334 | -0.469 | -0.2271 | No |
| 49 | STAT3 | na |  |  | 15037 | -0.567 | -0.2481 | Yes |
| 50 | A2M | na |  |  | 15146 | -0.588 | -0.2375 | Yes |
| 51 | HMOX1 | na |  |  | 15431 | -0.638 | -0.2347 | Yes |
| 52 | IFNGR2 | na |  |  | 15618 | -0.672 | -0.2259 | Yes |
| 53 | IL10RB | na |  |  | 15841 | -0.712 | -0.2178 | Yes |
| 54 | GRB2 | na |  |  | 15845 | -0.713 | -0.1983 | Yes |
| 55 | CBL | na |  |  | 16094 | -0.768 | -0.1900 | Yes |
| 56 | IFNAR1 | na |  |  | 16431 | -0.839 | -0.1844 | Yes |
| 57 | ITGB3 | na |  |  | 16433 | -0.839 | -0.1612 | Yes |
| 58 | STAT1 | na |  |  | 16812 | -0.929 | -0.1553 | Yes |
| 59 | IFNGR1 | na |  |  | 18491 | -1.701 | -0.1960 | Yes |
| 60 | IL6ST | na |  |  | 18809 | -2.032 | -0.1565 | Yes |
| 61 | IL13RA1 | na |  |  | 18844 | -2.080 | -0.1008 | Yes |
| 62 | CD9 | na |  |  | 18879 | -2.138 | -0.0435 | Yes |
| 63 | STAM2 | na |  |  | 18906 | -2.175 | 0.0153 | Yes |
Table: GSEA details [plain text format]

  

Fig 2: HALLMARK\_IL6\_JAK\_STAT3\_SIGNALING      
 Blue-Pink O' Gram in the Space of the Analyzed GeneSet

  

Fig 3: HALLMARK\_IL6\_JAK\_STAT3\_SIGNALING: Random ES distribution      
 Gene set null distribution of ES for **HALLMARK\_IL6\_JAK\_STAT3\_SIGNALING**
